# Piezoelectricity from Dopant-Induced Structural Distortions in Molecular Crystals Revealed by Raman Spectroscopy

**DOI:** 10.1021/jacs.6c06274

**Published:** 2026-07-10

**Authors:** Shiri Dishon Ben Ami, Noam Pinsk, Michal Hartstein, Shir Abrahami Ben Harush, Sergey Khodorov, Isabelle Weissbuch, Tevie Mehlman, Alexander Brandis, Meir Lahav, Igor Lubomirsky, Leeor Kronik, David Ehre, Omer Yaffe

**Affiliations:** † Department of Molecular Chemistry and Materials Science, 34976 Weizmann Institute of Science, Rehovoth 7610001, Israel; ‡ Department of Chemical and Biological Physics, Weizmann Institute of Science, Rehovoth 7610001, Israel; § Life Sciences Core Facilities, Weizmann Institute of Science, Rehovoth 7610001, Israel

## Abstract

Crystal engineering offers a route to functional molecular solids through controlled noncovalent interactions. In centrosymmetric crystals, piezoelectricity can be induced by stereoselective doping, where chiral additives are incorporated in a biased orientation that lowers inversion symmetry and generates net polarization. However, the microscopic origin of this polarization, specifically whether it arises primarily from dipole mismatch between the dopant and the host molecule it replaces, or from dopant-induced lattice distortion, remains generally unclear. Here, we disentangle these two contributions by incorporating four chiral *N*-acetyl-*L*-amino acids into the centrosymmetric crystal *N*-acetyl-*DL*-valine and examining the resulting materials using piezoelectric measurements, density functional theory (DFT), and low-frequency Raman spectroscopy that reveals concentration-dependent symmetry-breaking phenomena not resolved by conventional X-ray diffraction. We show that in the systems studied, macroscopic piezoelectricity correlates directly with the magnitude and orientation of local lattice distortions, whereas dipole mismatch along the polar axis plays a secondary role. These results establish a direct structure–function relation in doped molecular crystals and provide predictive design principles for engineering electromechanical response in molecular crystals through biased local distortions.

## Graphical abstract

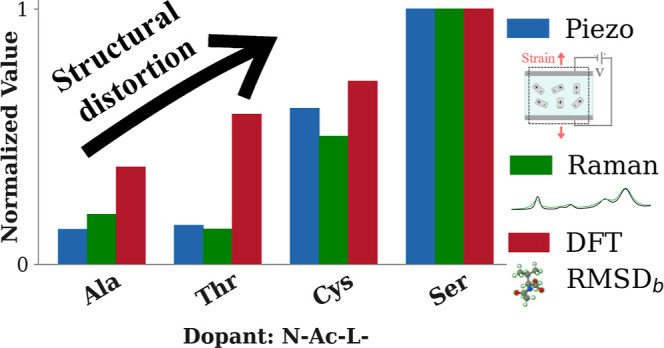

## Introduction

Crystal engineering of molecular crystals provides a conceptual framework for designing molecular solids through controlled solid-state assembly.^1,2^ Organic crystals are governed by a hierarchy of weak and tunable intermolecular interactions. This intrinsic modularity allows molecules to be treated as building blocks and their interactions to be viewed as design elements, allowing for rational control over crystal structure.^3–5^ Within this framework, deliberate reduction of crystal symmetry has emerged as a compelling strategy to control crystal properties such as polymorphism and polarization.^6–9^ Of the 32 crystallographic classes, only 10 are polar—these permit technologically relevant macroscopic properties such as piezoelectricity and pyroelectricity, which are forbidden in centrosymmetric crystals.^10,11^ Therefore, inducing symmetry breaking in nonpolar crystals could open new pathways to endow a much broader range of materials with such functional properties.

There are multiple pathways to achieve such symmetry lowering, including molecular substitution,^12^ solid solutions,^7^ and defect incorporation.^13^ Importantly, symmetry reduction does not need to involve a complete reconstruction of the host lattice. Subtle, biased perturbations that favor one orientation or distortion over its symmetry-related counterpart can be sufficient to generate a net polar axis and activate functional responses at the macroscopic scale.^14,15^ In many cases, dopant incorporation is polar, and because the dopant molecule is often larger than the host molecule, it induces a local structural distortion that can break inversion symmetry and generate polarization.^16,17^

A prominent approach to symmetry reduction is stereoselective doping,^18–21^ which exploits chiral molecules as dopants within centrosymmetric enantiomeric molecular crystals composed of alternating enantiomeric layers. In this process, dopant incorporation is governed by chiral surface recognition during crystal growth, whereby dopant molecules interact selectively with growth surfaces according to the handedness of the exposed layer. As schematically illustrated in Figure 1a, dopants with *L* chirality can be added to the crystals only into the *L*-layers. Therefore, they preferentially recognize and bind to growth sites associated with *D*-layers of the host crystal, and vice versa.^22^ When incorporated, a dopant molecule that differs from the host in size and dipole moment leads to two key contributions to polarization: one from the dipole mismatch and another from local structural distortion and strain in the host lattice.^16^ Understanding these contributions is crucial for establishing design principles for controlled polarization and piezoelectricity in doped molecular crystals.

**Figure 1:**
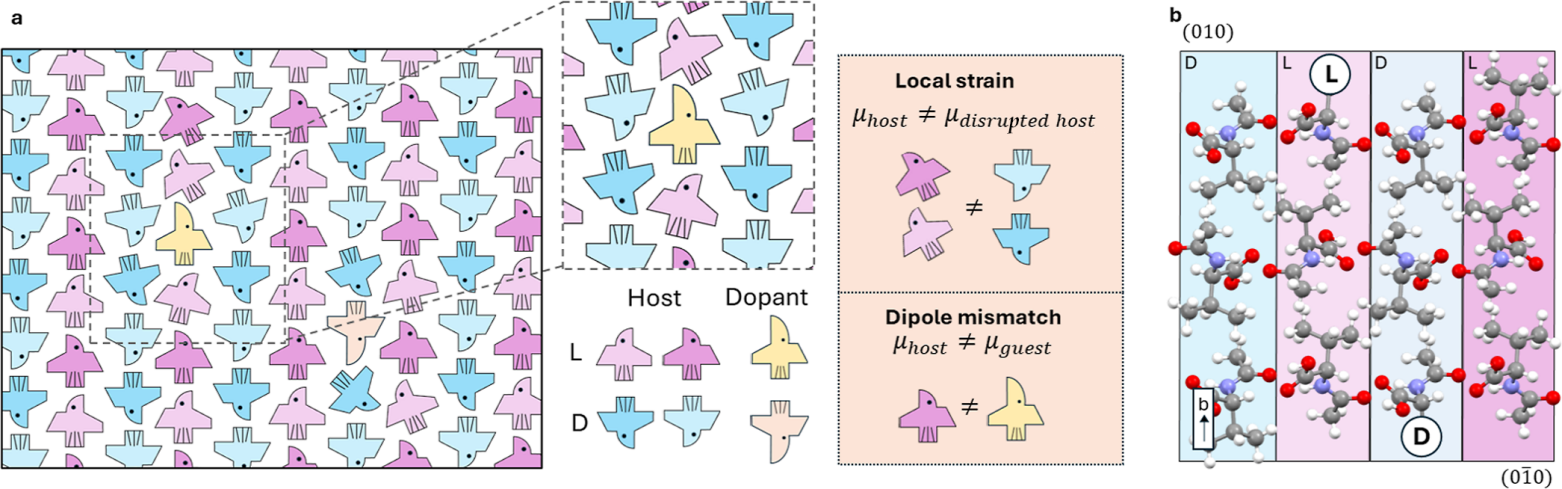
(a) Schematic illustration of stereoselective doping in centrosymmetric enantiomeric molecular crystals and its contributions to polarization. Two distinct contributions to the induced polarization upon dopant incorporation, namely, the dipole mismatch between the dopant and the host molecule it replaces, and the local structural distortion and strain imposed on the surrounding host lattice. (b) Enantiospecific surface recognition during crystal growth in a racemic crystal composed of alternating enantiomeric layers, where dopants of *L* chirality preferentially bind to growth sites associated with *D-*layers of the host crystal, and vice versa, leading to selective incorporation and symmetry reduction.

In this study, we address this challenge by systematically doping the centrosymmetric host crystal *N*-acetyl-*DL*-valine (*N*-Ac-*DL*-Val) with four chemically related chiral dopants and correlating structural, spectroscopic, and functional measurements. Using *N*-Ac-*DL*-Val as the host enables incorporation of dopants that are either smaller or larger than the host, allowing control over the magnitude of the deformation and the dipole mismatch between the host and the guest. We estimate the induced piezoelectric response for each dopant–host system and combine these measurements with Raman scattering and dispersion-augmented density functional theory calculations.^23,24^ Because conventional X-ray diffraction is insensitive to such small local distortions,^25^ we employ low-frequency Raman spectroscopy together with DFT calculations to probe the resulting structural perturbations. This integrated approach demonstrates the system of doped *N*-Ac-*DL*-Val as a case study, which suggests that the emergence of piezoelectricity correlates with an oriented distortion of the host lattice. This demonstrates that dopant-induced lattice deformation, rather than intrinsic dipole mismatch, is the dominant contributor to the piezoelectric response in this system.

## Results and Discussion

Figure 1b shows the structure of *N*-Ac-*DL*-Val, which crystallizes in a centrosymmetric monoclinic structure (*P*2_1_/*c*) composed of alternating *D* and *L* molecular layers stacked along the crystallographic *b* axis.^26^ This layered chiral architecture enables stereoselective doping through chiral surface recognition during crystal growth^21^ (see Section S1 in the Supporting Information). Consistent with this mechanism, *N*-acetyl-*L*-amino acids are selectively incorporated into one subset of enantiomeric layers in a polar orientation, generating a biased perturbation along the *b*-axis without reconstructing the structure of the host lattice.^16^ Throughout this work, *L*-chiral dopants were used to induce a net polarization by selectively occupying only one enantiomeric sublattice. This enantiospecific doping process reduces the symmetry of *N*-Ac-*DL*-Val crystals from (*P*2_1_/*c*) to (P2_1_), breaking centrosymmetry and inducing polarization along the *b*-axis.

The choice of dopants (Figure 2) was *N*-acetyl-*L*-serine (*N*-Ac-*L*-Ser), *N*-acetyl-*L*-cysteine (*N*-Ac-*L*-Cys), *N*-acetyl-*L*-threonine (*N*-Ac-*L*-Thr), and *N*-acetyl-*L*-alanine (*N*-Ac-*L*-Ala). This choice was designed to decouple the effects of molecular dipole mismatch from dopant-induced lattice distortion. Accordingly, two key factors were considered: (1) the dipole mismatch compared to the host molecule, *N*-Ac-*DL*-Val, projected along the *b*-axis in the relaxed state and (2) the local lattice distortion induced by the dopant in the relaxed state. Quantification of these factors was performed using DFT. Multiple initial dopant configurations within the host lattice were evaluated to identify stable relaxed structures for each dopant–host system. Relaxation was computed within the Perdew–Burke–Ernzerhof (PBE) exchange–correlation functional,^27^ augmented by D3 dispersion with the Becke–Johnson damping^28,29^ (more details in Section S1 of the Supporting Information). Figure 2 shows a zoom-in on the most stable DFT-optimized structures, illustrating the distinct local environments adopted by the host molecules around the dopant (see Figure S1 for full supercell). Less stable optimized structures are shown in Figures S2–S4.

**Figure 2:**
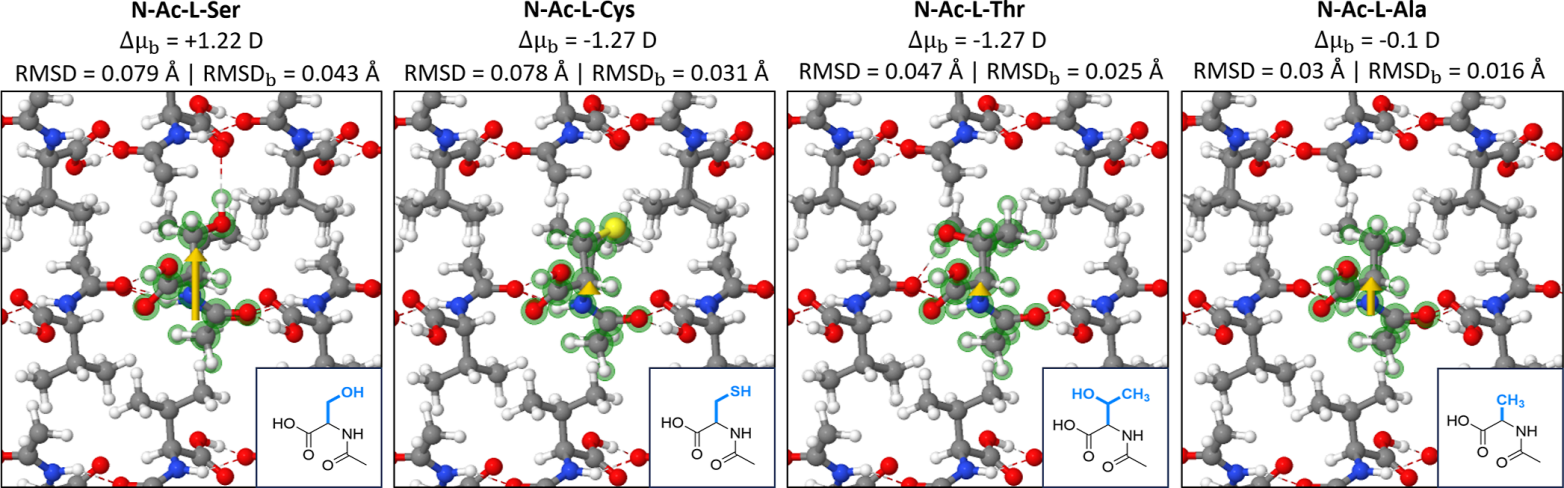
Zoom-in visualization of the most stable DFT-optimized conformations found for *N*-Ac-*DL*-Val doped with different dopants. Dopant atoms are highlighted with green halos, and the inset contains a schematic illustration of the dopant molecules. The orange arrows correspond to the *b*-components of the calculated dipole moments of the isolated dopant molecules in their crystal configuration, in Debye (D), and the difference in magnitude from the *b*-component dipole moment of the host molecule is indicated. The RMSD and RMSD_*b*_ of host atomic positions within a 10 Å radius from the dopant are indicated, quantifying the extent of local lattice distortion in the host molecules around the dopant. RMSD_*b*_ is projected onto the crystallographic *b*-axis, isolating the distortion along the direction in which piezoelectricity was measured. Atom colors: carbon, gray; oxygen, red; hydrogen, white; and nitrogen, blue.

The dipole mismatch was quantified by calculating the dipole moment of each isolated dopant molecule in its lowest-energy crystal configuration, projecting it along the crystallographic *b* axis, and comparing its magnitude to the *b*-component of the host molecule dipole moment. The host molecule, *N*-Ac-*DL*-Val, exhibits a dipole moment of 1.57 D along the *b* axis. Relative to this value, *N*-Ac-*L*-Ala shows a minimal dipole mismatch (Δμ_*b*_ = −0.10 D). In contrast, *N*-Ac-*L*-Thr/Cys/Ser exhibit a substantial dipole mismatch (Δμ_*b*_ = −1.27 D/–1.27 D/+1.22 D, respectively).

Dopant-induced structural perturbation was quantified by evaluating the extent of local lattice distortion, calculated as the root-mean-square deviation (RMSD) of the atomic displacements of host molecules surrounding each dopant

1 $$RMSD=\sqrt{\frac{1}{N}\sum_{i=1}^{N}|\Delta r_{i}{|}^{2}}$$

where Δ**r**_*i*_ is the Cartesian displacement of atom *i* between the pure and doped DFT-relaxed structures, and *N* is the number of host atoms, excluding dopant sites. We additionally report RMSD_*b*_, computed identically but replacing |Δ**r**_*i*_| with the scalar projection of Δ**r**_*i*_ onto the crystallographic *b*-axis, isolating the component of lattice distortion along the direction in which the piezoelectric response is measured. Both quantities were calculated within a 10 Å radius from the dopant center.

Applying this analysis to the different systems, *N*-Ac-*L*-Ala has a much smaller functional group than that of the *N*-Ac-*L*-Val molecule it replaces. It therefore features negligible steric perturbation with an RMSD of 0.03 Å and an RMSD_*b*_ of 0.016 Å. *N*-Ac-*L*-Thr closely matches the host molecule in size and shape, thereby minimizing dopant-induced lattice distortion, yielding an RMSD of 0.047 Å and an RMSD_*b*_ of 0.025 Å. Conversely, *N*-Ac-*L*-Cys and *N*-Ac-*L*-Ser introduce significant local lattice distortions due to differences in their functional groups compared to the host, resulting in a higher degree of structural perturbation with an RMSD of 0.078 Å and an RMSD_*b*_ of 0.031 Å for *N*-Ac-*L*-Cys, an RMSD of 0.079 Å and an RMSD_*b*_ of 0.043 Å for *N*-Ac-*L*-Ser.

The above results suggest that the selected dopants can be categorized into three types based on their dipole mismatch and induced lattice distortion: (i) *N*-Ac-*L*-Ala, representing minimal lattice distortion and minimal dipole mismatch; (ii) *N*-Ac-*L*-Thr, exhibiting small distortion but significant dipole mismatch; and (iii) *N*-Ac-*L*-Ser and *N*-Ac-*L*-Cys, which introduce substantial levels of both distortion and mismatch. To experimentally test this, pure and doped *N*-Ac-*DL*-Val single crystals were synthesized via slow evaporation from supersaturated solutions. By introducing between 5 and 30 wt % of these specific *L*-chiral dopants into the growth mixtures, lattice incorporations of up to 7 mol % were successfully achieved, later quantified using liquid chromatography–mass spectrometry (LC–MS) following extensive structural and functional testing (see Section S1 in the Supporting Information for more details).

The successful enantiospecific incorporation of these dopants is immediately visually evident through systematic changes in the macroscopic crystal morphology (Figure S6).^22^ In the *N*-Ac-*DL*-Val system, doped crystals develop a more flattened habit, with the {010} face becoming the dominant developed surface.^16^ Because this specific face dictates the stacking direction of the alternating enantiomeric layers along the crystallographic *b*-axis, its inhibited growth provides independent, macroscopic validation of the stereoselective dopant binding mechanism occurring at the crystal growth interface. However, despite the pronounced morphological changes induced by doping, both powder and single-crystal X-ray diffraction measurements reveal no detectable change in the average crystal symmetry, classifying pure and doped crystals within the same centrosymmetric space group, independent of dopant identity or concentration (Figure S7). This apparent discrepancy highlights the different structural information obtained from diffraction measurements, which probe the average crystal structure, and spectroscopic probes such as Raman scattering and time-domain THz spectroscopy, which are sensitive to local structure, as demonstrated^31–39^ in molecular crystals, pharmaceuticals, and perovskites.

In an ideal crystal, selection rules limit the allowed Raman modes to only Γ-point phonons and determine both the number and symmetry of the vibrational modes.^40–42^ When local symmetry is perturbed by dopant-induced lattice distortions, these selection rules locally relax:^43^ modes that are symmetry-forbidden (non-Γ-point phonons) or weak in the pristine structure can acquire finite intensity, phonon line widths broaden due to enhanced disorder and scattering, and the relative intensities of symmetry-related modes are modified. Low-frequency Raman scattering, therefore, provides a direct spectroscopic fingerprint of local symmetry reduction that may remain undetected by probes of the average structure, highlighting that functional properties in molecular crystals can be governed by locally symmetry-broken environments rather than by the average crystallographic symmetry alone.

Figure 3 presents unpolarized Raman spectra,^44^ collected from the (010) crystal face of pure and doped crystals in the frequency range below 100 cm^–1^, together with the DFT-calculated spectrum of the pure crystal. The experimental spectra were normalized to a host lattice mode at 786 cm^–1^ (Figure S8). See Section S1 for more experimental details. Crystals doped with *N*-Ac-*L*-Ser and *N*-Ac-*L*-Cys, which according to the DFT analysis in Figure 2 induce substantial local lattice distortions, exhibit pronounced spectroscopic changes, including enhanced low-frequency background intensity, phonon peak broadening, and modifications of relative peak intensities. These features are consistent with the relaxation of Raman selection rules and increased structural disorder. In contrast, crystals doped with *N*-Ac-*L*-Ala and *N*-Ac-*L*-Thr, predicted by DFT to introduce smaller distortions, display spectra that largely overlap with those of the pure crystal, indicating minimal perturbation of the lattice dynamics.

**Figure 3:**
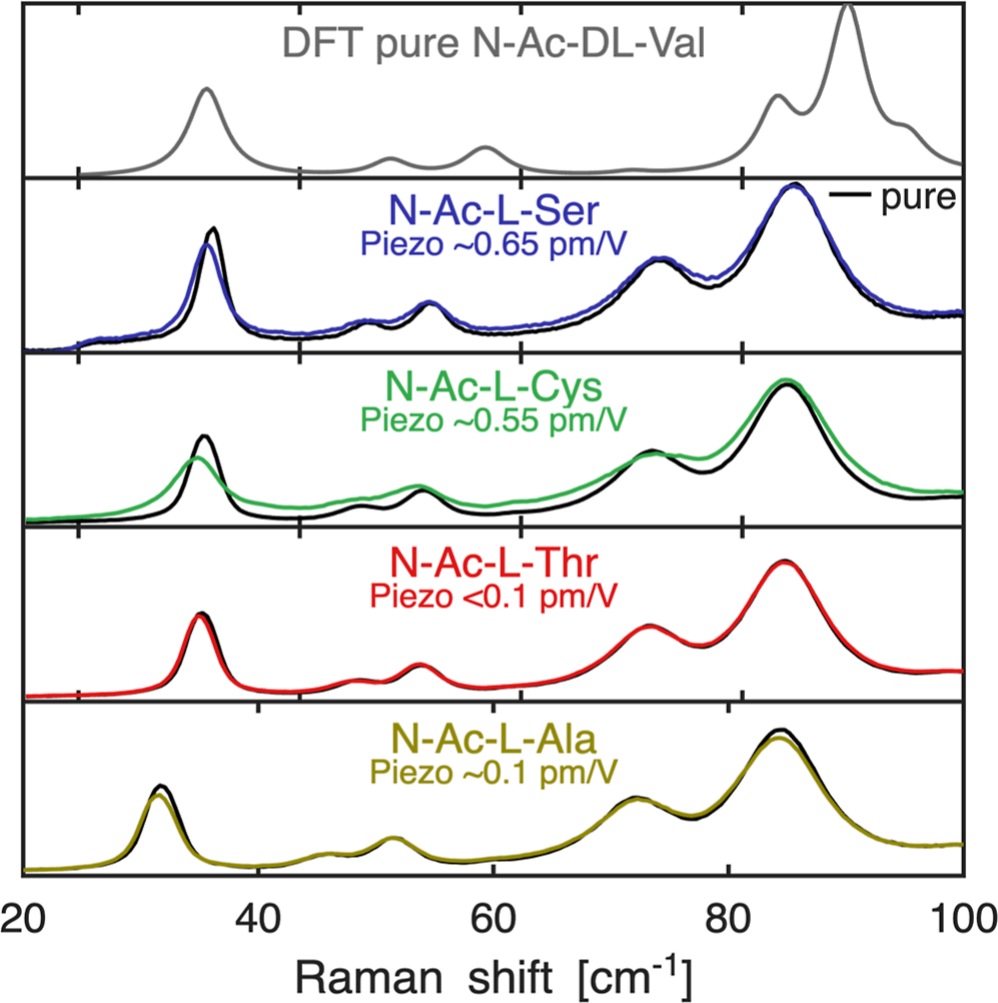
Low-frequency (20–100 cm^–1^) unpolarized Raman spectra of *N*-Ac-*DL*-Val crystals doped with 1.0 mol % *N*-Ac-*L*-Ser (blue), 6.7 mol % *N*-Ac-*L*-Cys (green), 3.6 mol % *N*-Ac- *L* -Thr (red), and 2.7 mol % *N*-Ac-*L*-Ala (yellow), overlaid on the Raman spectrum of the pure crystal (black), together with the DFT-calculated Raman spectrum of *N*-Ac-*DL*-Val at 0 K (gray).^30^ The piezoelectric response of crystals with a similar doping level is also noted.

Crucially, the spectroscopic signature of symmetry breaking maps directly onto the functional response: only *N*-Ac-*L*-Ser- and *N*-Ac-*L*-Cys-doped crystals display both pronounced low-frequency Raman signatures of symmetry reduction and a measurable piezoelectric effect. The piezoelectric response was quantified along the *b*-axis using Michelson interferometery^45,46^ and found to be up to 0.54 pm/V for 5 mol % *N*-Ac-*L*-Cys and up to 0.75 pm/V for 1 mol % *N*-Ac-*L*-Ser. For each dopant, at least ten crystals spanning a range of dopant concentrations were measured. Additionally, crystals doped with *N*-Ac-*L*-Cys and *N*-Ac-*L*-Ser showed a clear concentration-dependent piezoelectric response. In contrast, *N*-Ac-*L*-Ala-doped crystals exhibited only a weak effect, with an average piezoelectric coefficient of 0.12 ± 0.06 pm/V up to 3 mol %, without systematic dependence on concentration. An even smaller response was observed for *N*-Ac-*L*-Thr-doped crystals: samples that exhibited a detectable signal showed an average coefficient of 0.09 ± 0.06 pm/V up to 6 mol %, while others within the same concentration range showed no measurable response. Together, these results demonstrate that only dopants capable of inducing substantial local lattice distortion produce both excess low-frequency Raman intensity and a measurable piezoelectric response, establishing a direct link between local symmetry breaking, vibrational behavior, and macroscopic electromechanical functionality.

We now move beyond qualitative comparisons among different dopants and establish a direct quantitative relationship between dopant concentration, local symmetry perturbation reflected in the Raman line shape, and the emergence of macroscopic piezoelectricity. To that end, we focus on a single dopant, *N*-Ac-*L*-Cys, and examine its concentration dependence. *N*-Ac-*L*-Cys was selected because it can be incorporated at relatively high concentrations, and its distinct S–H stretching mode at 2575 cm^–1^ provides a direct spectroscopic marker of dopant incorporation.^47^ This approach allows one to track how incremental increases in dopant content modify the lattice vibrational response and the resulting piezoelectric activity. Figure 4a displays Raman spectra, normalized to a host molecular vibration at 2729 cm^–1^ (Figure S9), obtained from both pure and doped crystals with increasing *N*-Ac- *L* -Cys content. By integrating the S–H peak area (after subtracting the pure crystal background) and comparing it to LC–MS measurements, we confirm a direct correlation between the relative Raman intensities and the dopant’s molar concentration measured by LC–MS, as shown in Figure 4b. The integrated S–H intensities were averaged over three different spots along each crystal to account for spatial variations in dopant concentration, while LC–MS provides a bulk average over the entire crystal. We therefore attribute the observed nonlinear trend to local inhomogeneity in dopant incorporation. Furthermore, the S–H stretching vibration exhibits a strong polarization dependence, shown in Figure 4c (for more details, see Section S1 in the Supporting Information). This well-defined angular response proves that the dopant adopts a fixed orientation locked to the host lattice, thereby inducing a biased local strain field rather than random structural disorder.

**Figure 4:**
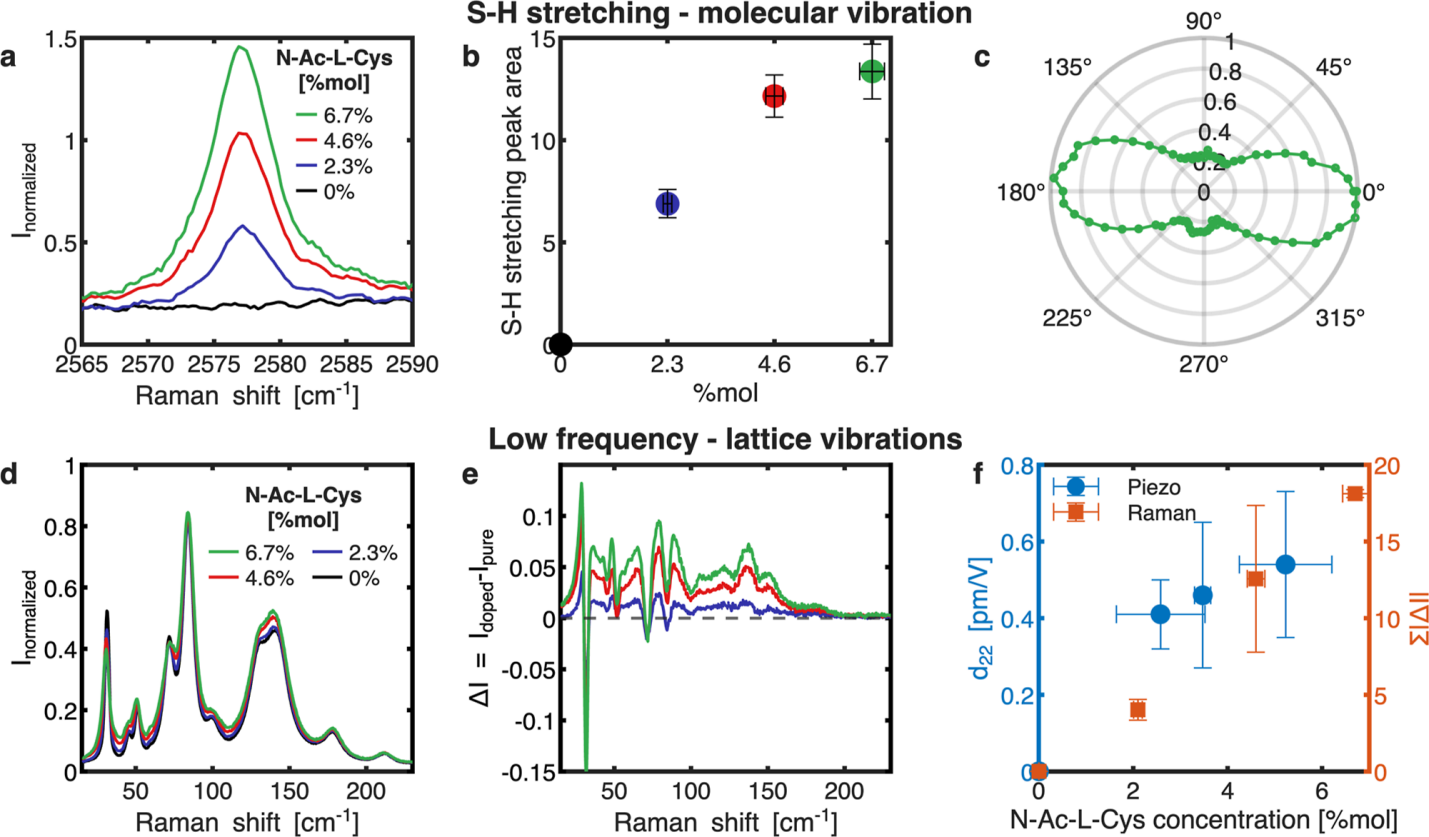
Effect of dopant concentration on the Raman and piezoelectric responses of *N*-Ac-*L*-Cys-doped *N*-Ac-*DL*-Val crystals. (a) Intensity of the S–H stretching vibration as a function of dopant concentration. (b) Correlation between the S–H peak area, averaged over three acquisitions from different locations on the crystal, and the dopant concentration determined by LC–MS. Error bars along the *x*-axis represent the LC–MS measurement uncertainty, while those along the *y*-axis correspond to the standard deviation of the integrated Raman intensities across the three acquisition points. (c) Polarization-dependent Raman intensity of the S–H mode, exhibiting a well-defined angular dependence consistent with a fixed dopant orientation within the host lattice. (d) Unpolarized low-frequency Raman spectra at increasing dopant concentrations, showing systematic enhancement of the low-frequency background and phonon peak broadening. (e) Differential low-frequency Raman spectra obtained by subtracting the spectrum of the pure crystal from that of the doped crystals, highlighting the excess intensity induced by symmetry breaking. (f) Correlation between dopant concentration and the piezoelectric coefficient (light blue circles) together with the integrated absolute differential low-frequency Raman intensity (orange squares), demonstrating a common concentration-dependent trend.

The structural consequences of this biased strain are evident in the low-frequency lattice vibrations. Figure 4d shows unpolarized low-frequency Raman spectra normalized to a host lattice mode at 786 cm^–1^ (Figure S10). As the dopant concentration increases, the spectra exhibit increased background intensity and broadened phonon peaks. These gradual changes in line shape reflect locally distorted environments that deviate from the pristine lattice symmetry responsible for discrete phonon modes. Subtracting the pure crystal baseline yields the differential low-frequency spectra in Figure 4e, which isolate and emphasize the excess intensity induced by this local symmetry breaking. Figure 4f establishes a direct link between these localized structural distortions and macroscopic electromechanical functionality. By integrating the absolute differential Raman intensity Σ­(|Δ*I*|) for the low frequencies $\left(<230cm^{-1}\right)$ , we observe a concentration-dependent structural deviation that closely matches the growth of the piezoelectric coefficient. Specifically, the piezoelectric response increases from 0.41 ± 0.09 pm/V at 2.58 ± 0.95 mol % to 0.54 ± 0.19 pm/V at 5.23 ± 0.98 mol %. We limit the calculation of Σ­(|Δ*I*|) to below 230 cm^–1^ (Figures S8 and S10) because only this range includes the information on the lattice modes.^40,48^

A quantitative comparison across different dopants highlights that, despite the dominance of local distortion, other aspects of the dopant strongly affect the piezoelectric response. Specifically, *N*-Ac-*L*-Ser and *N*-Ac-*L*-Cys, which exhibit the strongest piezoelectric responses upon doping, reach different maximum dopant concentrations during crystallization: up to 1 mol % for *N*-Ac-*L*-Ser and up to 7 mol % for *N*-Ac-*L*-Cys. Using the Raman spectra and piezoelectric values of the highest-doped crystals shown in Figure 3, we normalized both the piezoelectric response and Σ­(|Δ*I*|) by the corresponding dopant concentration. This comparison, shown in Figure S11, yields a consistent trend, with *N*-Ac-*L*-Ser exhibiting the largest concentration-normalized piezoelectric response and Σ­(|Δ*I*|), followed by *N*-Ac-*L*-Cys. Despite its smaller absolute Σ­(|Δ*I*|), *N*-Ac-*L*-Ser exhibits a larger overall piezoelectric response than *N*-Ac-*L*-Cys. These results indicate that dopant identity influences not only the extent of incorporation into the host lattice but also the magnitude of the local distortion that affects macroscopic piezoelectricity, as also reflected by the RMSD_*b*_.

Altogether, the above results demonstrate that piezoelectricity arises predominantly from biased local structural distortions that disrupt inversion symmetry rather than from the inherent dipole moment mismatch between the guest molecule and the host it replaces. It is important to clarify the relationship between the local nature of the analysis presented here and the macroscopic character of the measured piezoelectric response. In conventional piezoelectric materials, the electromechanical response often arises from collective, periodic distortions of a noncentrosymmetric lattice that are inherently delocalized. The present system operates via a fundamentally different mechanism. At the low doping concentrations employed here, individual dopant molecules are well separated. Indeed, the DFT-computed structural relaxation confirms that lattice distortions decay rapidly with distance from the dopant site: host molecules beyond the first coordination shell are essentially unperturbed and recover the geometry of the pure crystal. Each dopant therefore acts as an independent localized source of symmetry breaking. The macroscopic piezoelectric response is constructed from the accumulation of local contributions. In the present system, this accumulation is biased, rather than random, as supported by two complementary claims: the stereoselective incorporation mechanism determines where each dopant enters—exclusively into the same enantiomeric sublattice—while the DFT-calculated lowest-energy conformation suggests that the majority of dopant sites adopt a similar local geometry and orientation. Together, these constraints suggest that dopant-induced local distortions are preferentially oriented in the same direction, such that their contributions add up constructively. This is directly supported experimentally by the polarization-dependent Raman measurements of the S–H stretching mode, which demonstrate that the dopant population adopts a well-defined preferred orientation locked to the host lattice. To further validate the dipole mismatch quantification, we performed Berry phase calculations of the polarization along the *b*-axis for the most stable DFT-relaxed configurations of all four dopants (Figure S5). We note that the Berry phase values do not quantitatively match the isolated molecule dipole mismatches, which is expected because the solid-state polarization also contains contributions from the distortion of the surrounding host lattice. Nevertheless, the consistent trends across dopants indicate that the dominant contribution originates from the molecular replacement itself, validating the isolated molecule dipole projection as a meaningful estimate. Notably, *N*-Ac-*L*-Thr exhibits a substantial dipole mismatch by either measure yet shows a low piezoelectric response experimentally, suggesting that dipole mismatch alone is insufficient to drive macroscopic piezoelectricity and that dopant-induced lattice distortion is a governing factor in this system.

## Conclusions

In conclusion, we elucidate the structural origin of piezoelectricity in stereoselectively doped molecular crystals, using *N*-Ac-*DL*-Val as a model host system. By systematically decoupling dipole mismatch along the relevant crystallographic axis from local steric perturbations, we show that in this system macroscopic polarization is governed primarily by biased, dopant-induced lattice distortions rather than by dipole mismatch alone. Although such a mechanism could perhaps be anticipated from the structural perturbations introduced by dopants, our results provide direct experimental evidence linking dopant-induced lattice distortions to the resulting piezoelectric response. These results demonstrate how local structural perturbations can dominate the piezoelectric response even when dipole mismatch is minimized or reversed. We further demonstrate that low-frequency Raman spectroscopy provides a sensitive, quantitative, and broadly applicable probe of local symmetry breaking and strain fields that remain elusive to techniques sensitive only to the average structure, such as X-ray diffraction. Together, these results establish clear structure–function relationships between dopant-induced lattice distortions and piezoelectric response and provide practical design principles for activating and optimizing electromechanical functionality in doped molecular crystals through controlled solid-state assembly.

## Supplementary Material



## Notes

Materials and methods; DFT-doped structures: supercell of *N*-Ac- *DL* -Val with one dopant molecule; DFT-optimized structures of *N*-Ac- *DL* -Val doped with *N*-Ac- *L* -Cys, *N*-Ac- *L* -Ser and *N*-Ac- *L* -Thr; DFT-calculated dipole moments; structural CIF files of DFT-relaxed most stable doped configurations; photographs of pure and doped *N*-Ac- *DL* -Val crystals; powder X-ray diffraction (XRD) patterns in the logarithmic scale of pure *N*-Ac- *DL* -Val; low-frequency Raman spectrum of the four doped crystals, with respect to the pure spectrum, extended to a range of 15–800 cm^–1^; high-frequency Raman spectrum of the SH stretching vibration extended to a range of 2450–2800 cm^–1^; and low-frequency Raman spectrum of the *N*-Ac- *L* -Cys-doped crystals at various concentrations, extended to a range of 15–800 cm^–1^ (PDF)

## Abbreviations

DFT: Density Functional Theory
*N*-Ac-*DL*-Val: *N*-Acetyl- *DL* -Valine
*N*-Acetyl- *L* -Serine: *N*-Ac- *L* -Ser
*N*-Acetyl- *L* -Cysteine: *N*-Ac- *L* -Cys
*N*-Acetyl- *L* -Threonine: *N*-Ac- *L* -Thr
*N*-Acetyl- *L* -Alanine: *N*-Ac- *L* -Ala
PBE: Perdew–Burke–Ernzerhof
RMSD: Root-Mean-Square Deviation
LC–MS: Liquid Chromatography–Mass Spectroscopy;
