# Supplementary material for: Piezoelectricity from Dopant-Induced Structural Distortions in Molecular Crystals Revealed by Raman Spectroscopy
Source: J Am Chem Soc. 2026 Jul 10;148(28):30029–36. doi: 10.1021/jacs.6c06274 (PMC13397563; doi:10.1021/jacs.6c06274)
Supplement: Supplementary file 1 [file ja6c06274_si_001.pdf]

Piezoelectricity from Dopant-Induced Structural Distortions in Molecular Crystals Revealed by Raman Spectroscopy

Shiri Dishon Ben Ami,[1,†] Noam Pinsk,[2,†] Michal Hartstein,[1,†] Shir Abrahami Ben Harush,[1] Sergey Khodorov,[1] Isabelle Weissbuch,[1] Tevie Mehlman,[3] Alexander Brandis,[3] Meir Lahav,[1] Igor Lubomirsky,[1,*] Leeor Kronik,[1,*] David Ehre,[1,*] and Omer Yaffe[2,*]

[1]*Department of Molecular Chemistry and Material Science, Weizmann Institute of Science, Rehovot 76100, Israel*

[2]*Department of chemical and biological physics, Weizmann Institute of Science, Rehovot 76100, Israel*

[3]*Life Sciences Core Facilities, Weizmann Institute of Science, Hertzel 234, Rehovot, 7610001 Israel*

[†] These authors contributed equally to this work.

[*] Igor.Lubomirsky@weizmann.ac.il

[*] leeor.kronik@weizmann.ac.il

[*] David.Ehre@weizmann.ac.il

[*] Omer.Yaffe@weizmann.ac.il

Contents

S1 Materials and Synthesis **3**

A Crystal Growth 3

B Computational Methods 3

1 Calculated Doped Structures 3

2 Evaluating Host Deformations 3

3 Polarization Calculations 4

4 Calculated Raman Spectrum 4

C Liquid Chromatography Mass Spectrometry (LC–MS) for Compositional Characterization 4

D Raman Spectroscopy 5

E Piezoelectricity Measurements 5

S2 DFT Calculations **7**

A Supercell of N-Ac-DL-Val with one Dopant Molecule 7

B DFT Optimized Structures of N-Ac-DL-Val Doped with N-Ac-L-Cys 8

C DFT Optimized Structures of N-Ac-DL-Val Doped with N-Ac-L-Ser 9

D DFT Optimized Structures of N-Ac-DL-Val Doped with N-Ac-L-Thr 10

E DFT Calculated Dipole Moments 11

S3 Supplementary Figures **12**

References **18**

S1. Materials and Synthesis

A. Crystal Growth

A supersaturated solution (130%) of N-Ac-DL-Val (Sigma Aldrich, ≥99.0% (acidimetric)) with 5–30 wt% dopants was prepared by dissolving the powder in a solution of 90:10 ethyl acetate (Gadot) and ethanol (70%, Gadot), respectively. The solution was then heated to 40°C to achieve complete dissolution. The solutions were filtered through cotton wool into glass crystallization dishes, which were covered with perforated Al foil to allow slow evaporation in a cleanroom environment. The transparent single crystals were washed in ethyl acetate and dried. The doped crystals were grown in the presence of 5–30 wt% of the following dopants: N-Ac-L-Cys (Alfa Aesar, 98+ %), N-Ac-L-Ser (Biosynth), N-Ac-L-Ala (Biosynth), and N-Ac-L-Thr (10x Chem LLC, ≥97%).

B. Computational Methods

All solid-state calculations were performed within the framework of density functional theory (DFT), using the Vienna Ab initio Simulation Package (VASP), version 6.3.0[1]. The Perdew–Burke–Ernzerhof (PBE) exchange–correlation functional[2] was employed in combination with the D3 dispersion correction with Becke–Johnson damping[3,4]. A convergence threshold of 10^{-6} eV per cell was applied for the total energy, and 10^{-3} eV/Å for the residual forces. The plane-wave cutoff energy was set to 1000 eV.

1. Calculated Doped Structures

A $2 \times 2 \times 2$ supercell of N-Ac-DL-Val was constructed and relaxed using fixed lattice parameters: $a = 13.25$ Å, $b = 26.004$ Å, $c = 20.06$ Å, $\alpha = 90°$, $\beta = 105.37°$, and $\gamma = 90°$. The supercell contains 32 molecules, with one N-Ac-DL-Val molecule replaced by a dopant (see Figure S1), corresponding to a doping concentration of approximately 3%, comparable to the experimental values. All doped structures were relaxed with fixed lattice parameters using a single k-point for Brillouin zone sampling.

2. Evaluating Host Deformations

To quantify dopant-induced distortions of the host molecules, the root-mean-square deviation (RMSD) of atomic positions was calculated for each relaxed structure. Com-

parisons were performed between the relaxed pure host structure and the corresponding relaxed doped structure, excluding the replaced host molecule and the dopant molecule. In addition to the total RMSD over the entire cell, a localized RMSD within a 10 Å radius from the dopant center was computed to assess structural deformation in the vicinity of the dopant.

3. Polarization Calculations

The dipole moments of isolated dopant molecules were calculated in their orientation inside their optimized crystal structures, using the Q-CHEM code[5], employing the PBE functional[2] and the 6-31G* basis set. Macroscopic dipole moments of the doped crystals were calculated using the Berry phase formalism within the Modern Theory of Polarization.[6]

4. Calculated Raman Spectrum

Full atomic relaxations were performed starting from the experimentally determined structure of N-Ac-DL-Val, using a $3 \times 2 \times 2$ k-point grid for Brillouin zone sampling. Phonon frequencies were calculated using the finite-differences method with symmetry, employing atomic displacements of 0.01 Å. Isotropic Raman intensities were computed using the method of Porezag and Pederson[7], as implemented in the `vasp_raman_py`[8] Python code. This approach uses VASP to calculate the macroscopic dielectric tensor associated with each phonon mode. To simulate experimental broadening, a Lorentzian broadening of 2 cm^{-1} was applied to the calculated spectra.

C. Liquid Chromatography Mass Spectrometry (LC–MS) for Compositional Characterization

The samples were analyzed by LC–MS/MS using a UPLC Acquity I-Class system coupled with a Xevo TQ-XS mass spectrometer (both Waters). For the separation, an HSS T3 UPLC column (2.1 × 150 mm, 1.7 μm, Waters) was used with acidified (0.1% formic acid) acetonitrile in water as the mobile phase: 5% acetonitrile for 0.8 min, followed by a gradient to 95% acetonitrile over 4.8 min at a flow rate of 0.3 mL min^{-1}.

Mass spectrometry was performed using electrospray ionization in negative mode. Multiple reaction monitoring (MRM) parameters were optimized for each amino acid using the

corresponding standards (collision energy, eV): 130 → 88 (9) *m/z* for N-acetyl-L-alanine, 146 → 115.9 (9) and 146 → 73.9 (12) *m/z* for N-acetyl serine, 158 → 116 (12) *m/z* for N-acetyl-L-valine, 159.8 → 115.8 (9) and 159.8 → 73.7 (12) *m/z* for N-acetyl-L-threonine, 162 → 84 (9) *m/z* for N-acetyl-L-cysteine, and 323 → 193.8 (12), 323 → 161.8 (15), 323 → 127.8 (21), and 323 → 115.8 (30) *m/z* for N,N′-diacetyl cystine.

Quantification was based on standard curves of an amino acid mixture in the concentration range 0.01–10 μM using TargetLynx software.

D. Raman Spectroscopy

Raman measurements were performed using a custom-built apparatus consisting of a 1 m long dispersive Raman spectrometer (Horiba FHR-1000) equipped with interchangeable gratings (600, 1800, or 2400 mm^{-1}). The optical path included notch filters to enable access to the low-frequency Raman region (> 10 cm^{-1}). All samples were measured in a back-reflected geometry using a $10\times$ objective. Excitation was provided by a 532 nm Nd:YAG Prometheus laser or a 785 nm Toptica diode laser. The laser power was maintained below 12 mW to ensure sufficient signal intensity while avoiding sample heating. For the polarization-dependent measurement, a half-wave plate was used to scan the Raman response to a polarized laser beam across $360°$ at an angular resolution of 5°. A polarizer was placed in the downstream of the optical setup, collecting only the polarization component parallel to the optical table. By tracing the maximum intensity of the SH stretching vibration peak, we extracted the polarization dependence of the mode, as shown in Figure 4c in the main text.

E. Piezoelectricity Measurements

Piezoelectric measurements were performed using a Michelson-type interferometer. The top and bottom electrodes of the samples were fabricated using conductive silver paint. A mirror (glass coated with a 300 nm aluminum layer) was affixed to the top of each sample using silver paint. An external voltage in the range of 50–200 V was applied to the crystal via an amplified sine-wave signal (Trek) generated by a function generator (Rigol). In parallel, a 632.8 nm laser beam was directed onto a beam splitter. One beam was reflected from the mirror attached to the sample, while the other was reflected to-

ward a displacement mirror. The two beams were then recombined and directed onto a photodetector.

The displacement of the sample was determined by analyzing oscillations of the target signal within the carrier interference signal. Specifically, the target maxima were identified at the quadrature bias point of the carrier interference signal, corresponding to the point of maximum derivative of the transfer function, located between constructive and destructive interference. Due to mechanical instabilities in the environment, a one-axis moving mirror was employed to record all states of the response signal. The interferometer consists of two perpendicular arms that generate interference: one containing the displacement mirror and the other containing the measured sample. The photodetector output was amplified using a transimpedance amplifier, and the phase was determined using a lock-in amplifier. The displacement response to the applied voltage was measured over a frequency range of 200–800 Hz. One or two frequencies were selected, and the displacement as a function of voltage was recorded. The slope of this curve was used to determine the piezoelectric coefficient d_{22}. The reported value corresponds to an average over measurements performed on 5–10 crystals of each type.

S2. DFT Calculations

A. Supercell of N-Ac-DL-Val with one Dopant Molecule

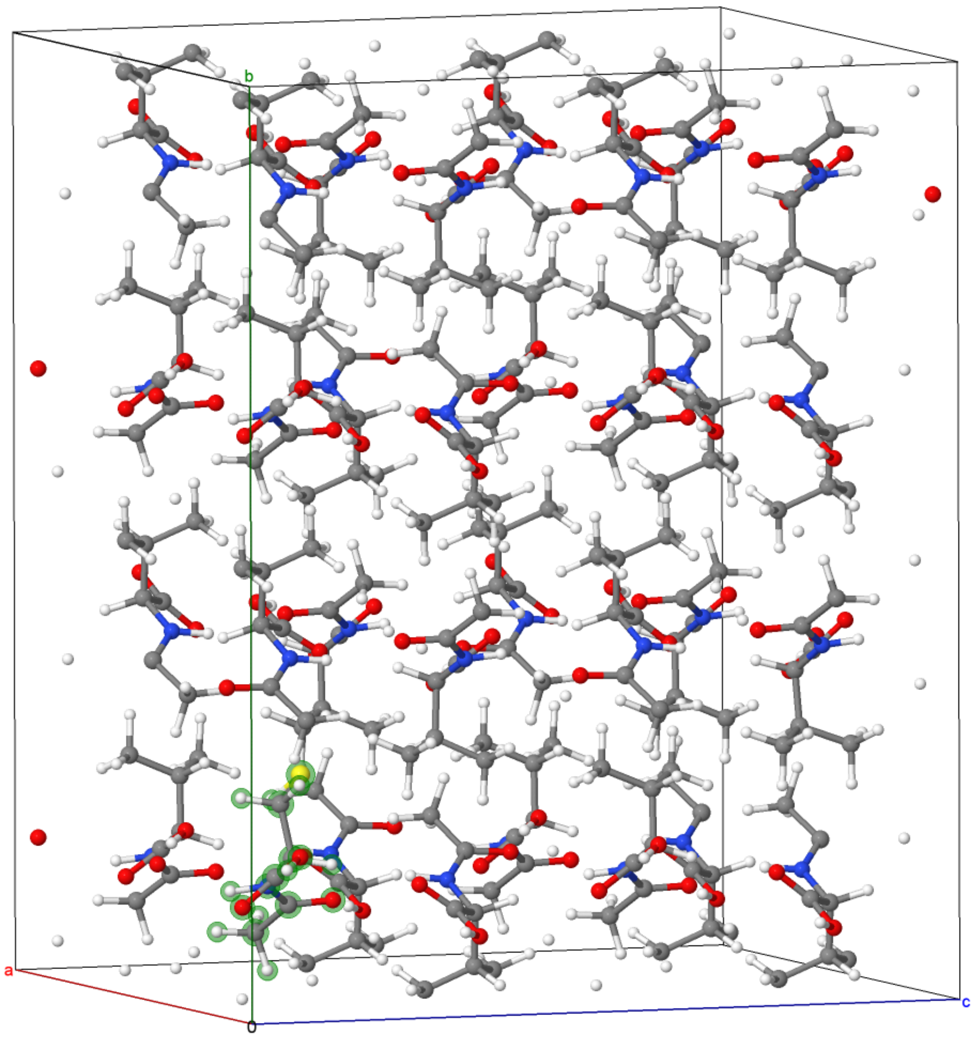

Figure S1: A supercell containing 31 host molecules of N-Ac-DL-Val and one molecule of dopant, corresponding to a doping concentration of approximately 3%. Dopant atoms are highlighted with green halos. Lattice parameters: a = 13.25 Å, b = 26.004 Å, c = 20.06 Å, α = 90°, β = 105.37°, and γ = 90°. A similar cell with the same dimensions was used for all DFT doped structures optimization calculations.

B. DFT Optimized Structures of N-Ac-DL-Val Doped with N-Ac-L-Cys

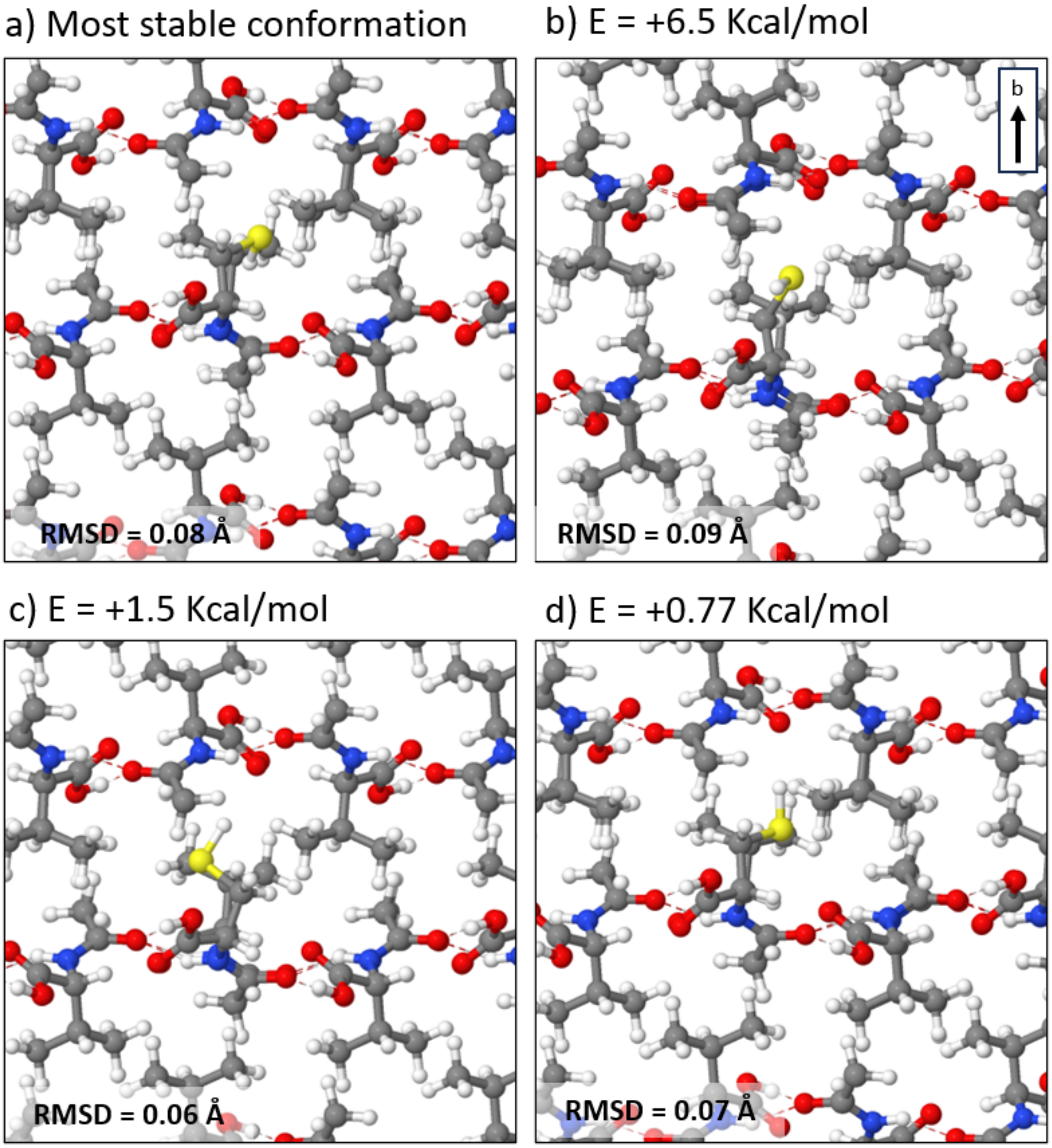

Figure S2: Four lowest-energy conformations found for N-Ac-DL-Val doped with N-Ac-L-Cys. a) Most stable conformation, as shown in the main text, and b-d) three less stable conformations. Each figure indicates the relative energy compared to the most stable configuration, as well as the computed RMSD of the host's atomic positions compared to the pure crystal, within a 10 Å range from the dopant center.

C. DFT Optimized Structures of N-Ac-DL-Val Doped with N-Ac-L-Ser

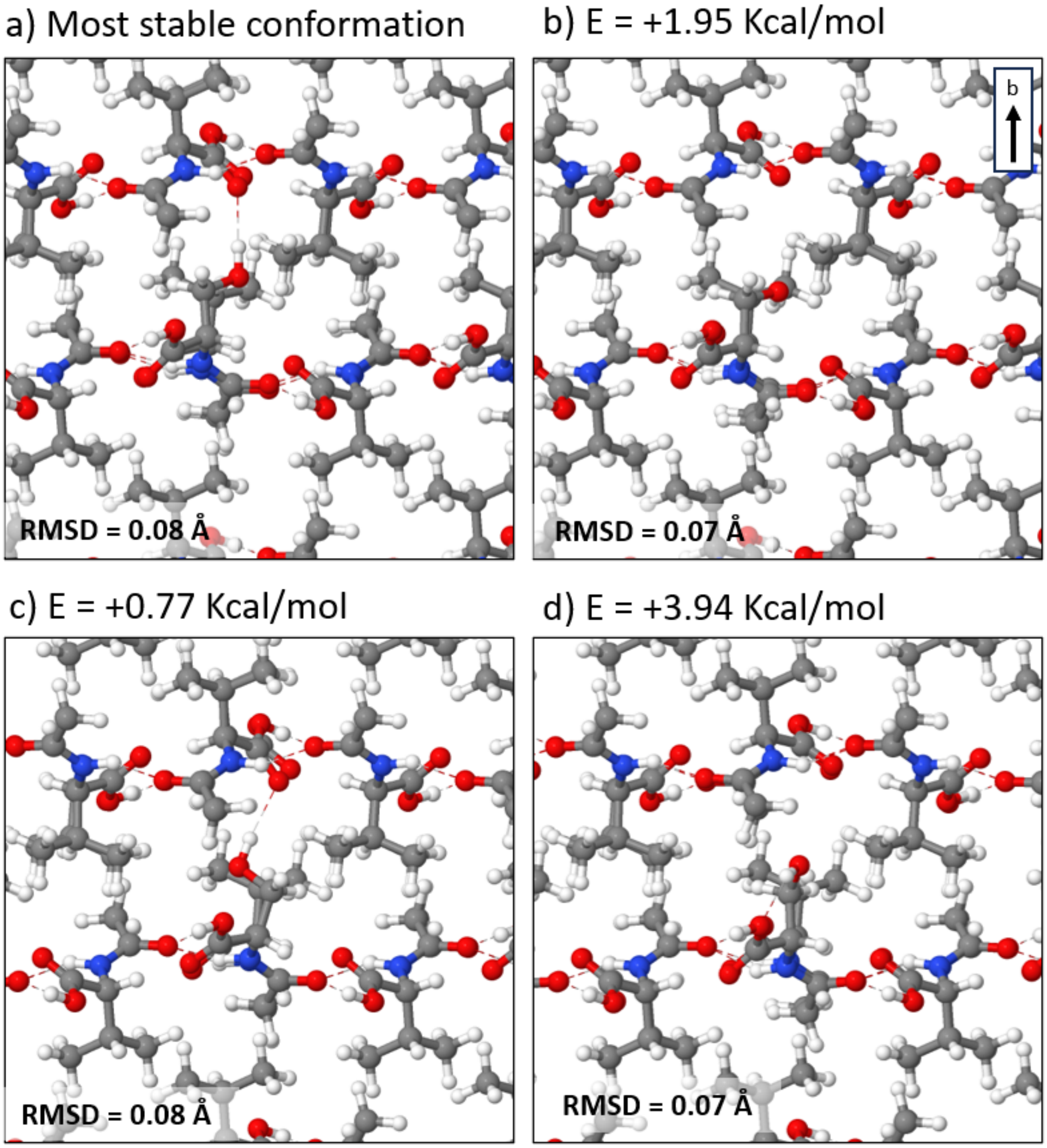

Figure S3: Four lowest-energy conformations found for N-Ac-DL-Val doped with N-Ac-L-Ser. a) Most stable conformation, as shown in the main text, and b-d) three less stable conformations. Each figure indicates the relative energy compared to the most stable configuration, as well as the computed RMSD of the host's atomic positions compared to the pure crystal, within a 10 Å range from the dopant center.

D. DFT Optimized Structures of N-Ac-DL-Val Doped with N-Ac-L-Thr

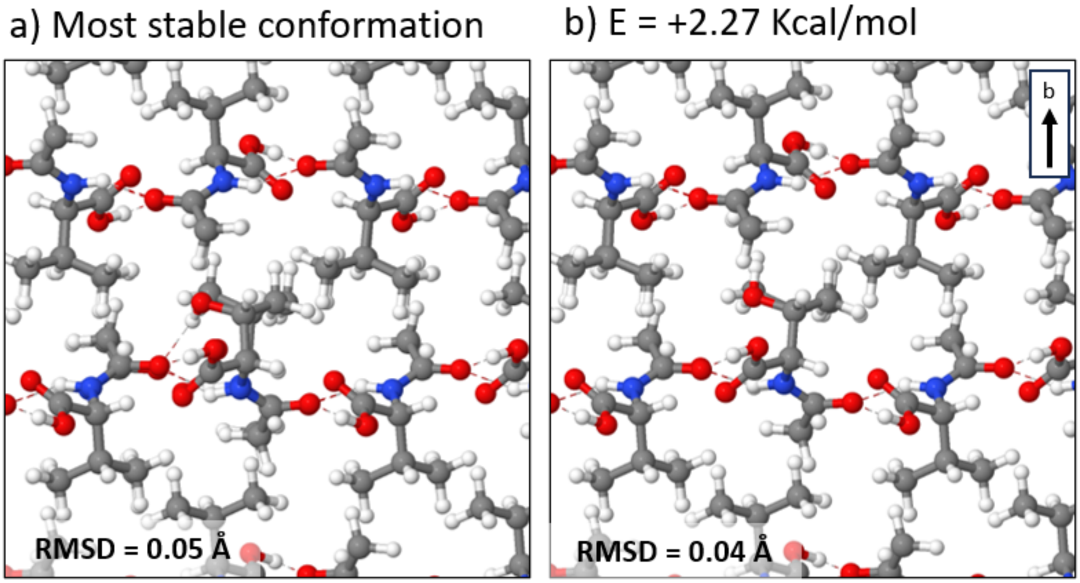

Figure S4: Two lowest-energy conformations found for N-Ac-DL-Val doped with N-Ac-L-Thr. a) Most stable conformation, as shown in the main text, and b-d) three less stable conformations. Each figure indicates the relative energy compared to the most stable configuration, as well as the computed RMSD of the host's atomic positions compared to the pure crystal, within a 10 Å range from the dopant center.

E. DFT Calculated Dipole Moments

The macroscopic polarization along the crystallographic b-axis was computed for the most stable DFT-relaxed doped supercell of each dopant, using the Berry phase method.[6] The total polarization was obtained by summing the ionic and electronic contributions along b, calculated for the pure and doped supercells. As expected, the pure crystal yields zero net polarization along b, consistent with its centrosymmetric space group. Since Berry phase polarization is defined only up to a polarization quantum $n \cdot B$, where B is the lattice parameter along b and n is an integer, we selected the branch that minimizes the magnitude of the computed polarization, as this choice is free of assumptions about the absolute polarization. The resulting comparison between $\Delta\mu_b$ and P_b is shown in Figure S5.

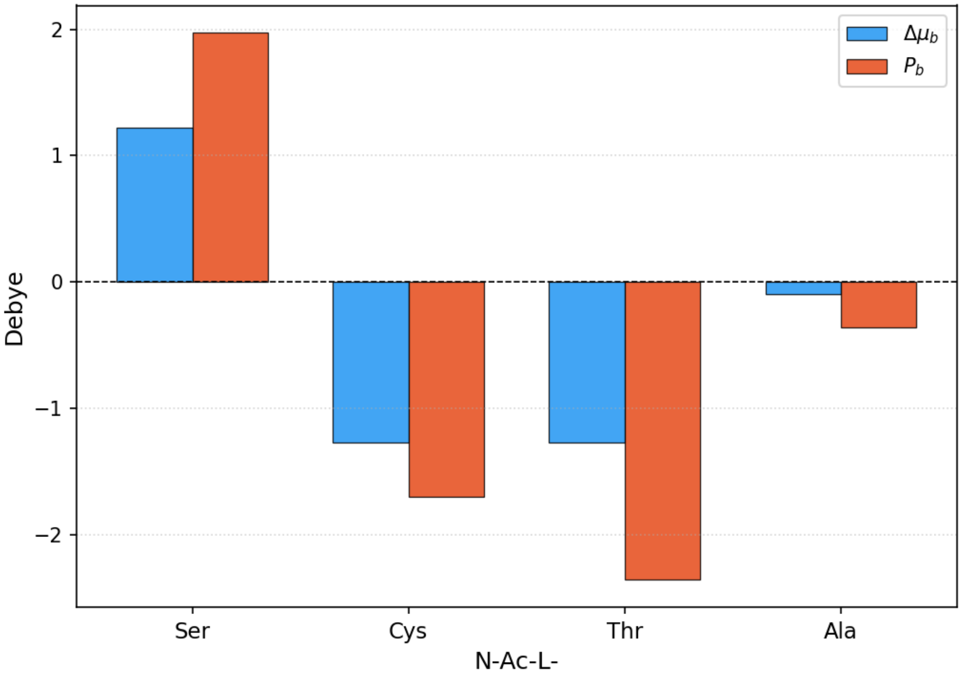

Figure S5: Comparison of two DFT-calculated dipole moments along the crystallographic b-axis for each dopant. Blue bars show the dipole mismatch $\Delta\mu_b$, which is the difference between the b-component of the dipole moment of the isolated dopant molecule (in its most stable DFT-predicted crystal configuration) and that of the isolated host molecule (*N*-Ac-*DL*-Val). Orange bars show P_b, the change in macroscopic polarization along b upon doping, calculated via the Berry phase method for the most stable DFT-relaxed doped supercell.

S3. Supplementary Figures

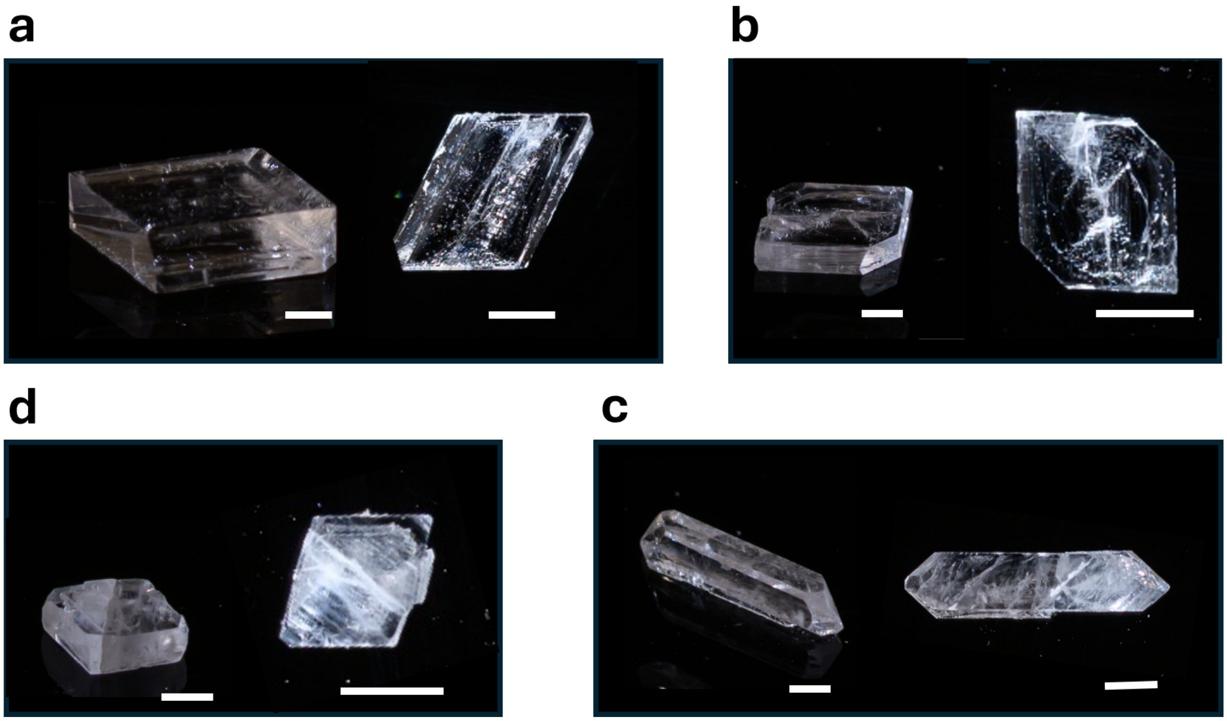

Figure S6: Photographs of pure and doped N-Ac-DL-Val crystals: (a) pure, (b) doped with N-Ac-L-Cys, (c) doped with N-Ac-L-Ser, and (d) doped with N-Ac-L-Ala. Scale bar: 2 mm.

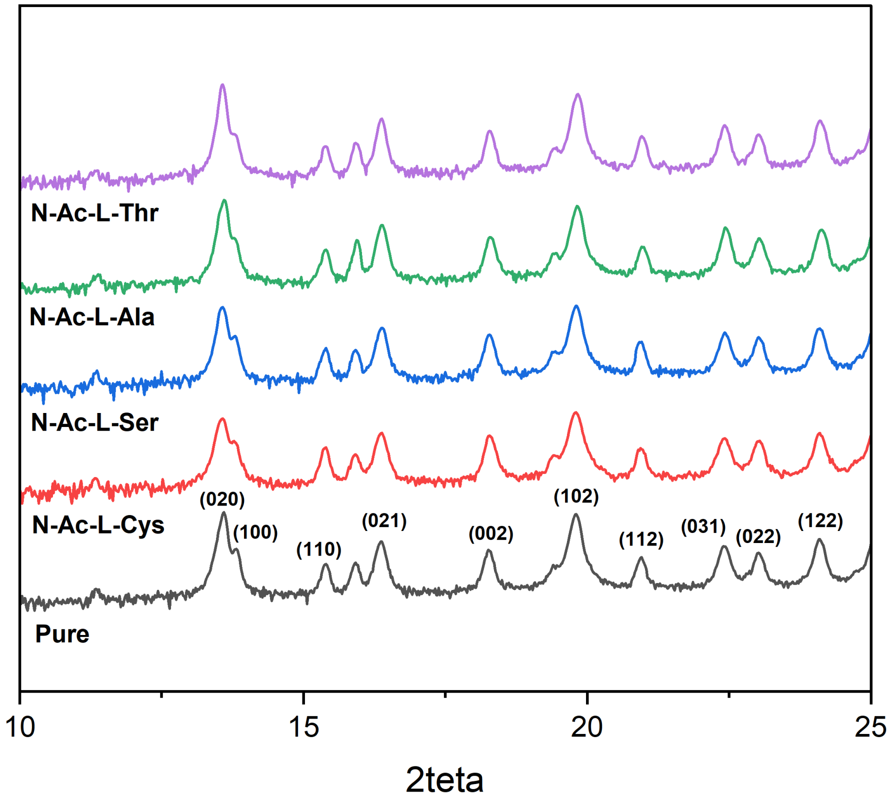

Figure S7: Powder X-ray diffraction (XRD) patterns in logaritic scale of pure N-Ac-DL-Val (black) and doped N-Ac-L-Cys (red), N-Ac-L-Ser (blue), N-Ac-L-Ala (green) and N-Ac-L-Thr (purple) crystals. Powder XRD patterns of doped crystals likewise show no detectable change in the crystal structure compared to the pure material.

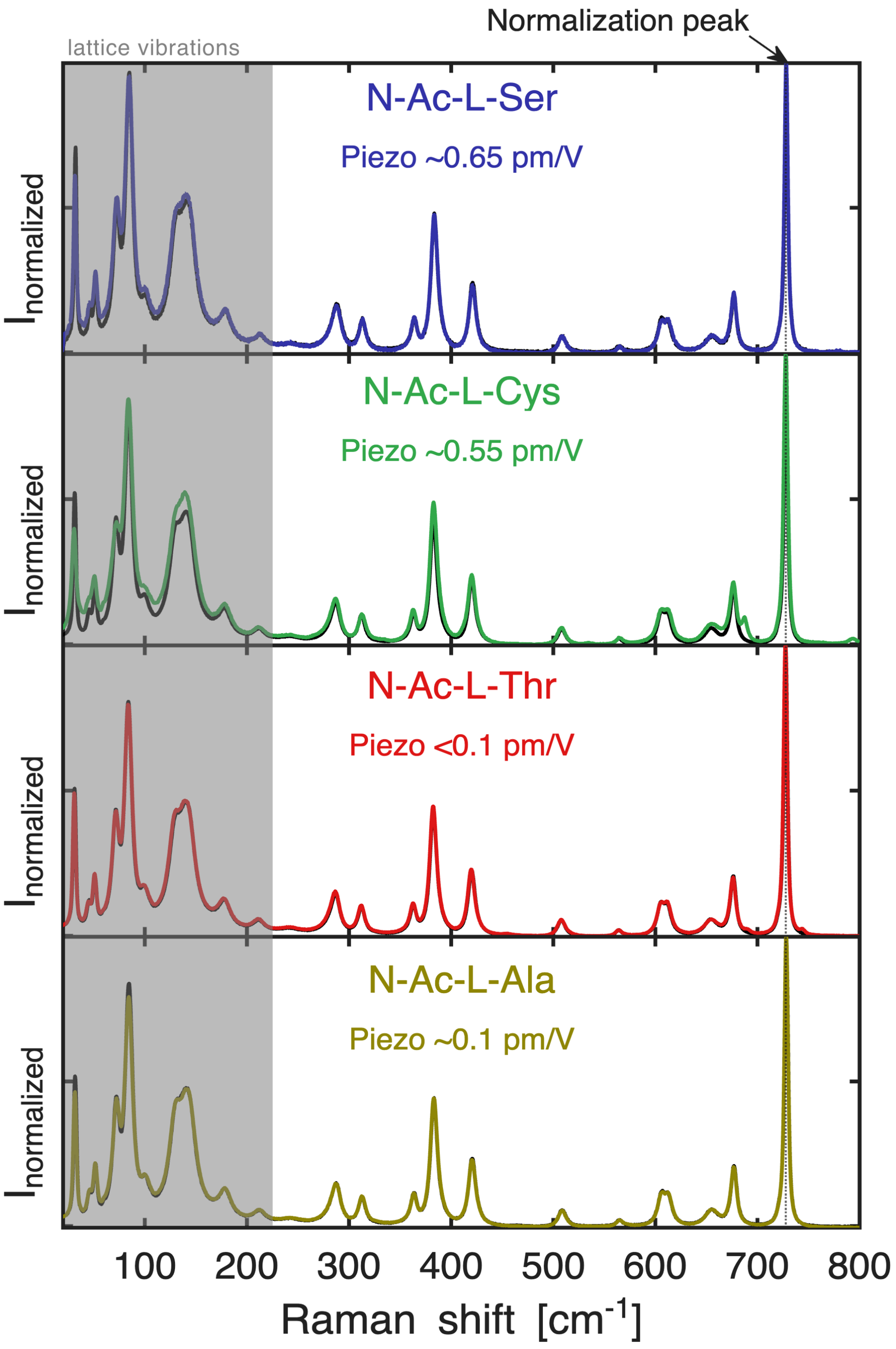

Figure S8: Raman spectrum of the four doped crystals, with respect to the pure spectrum, between 15 – 800 cm^{-1} demonstrating the high-frequency peak all spectra were normalized to. Grey shaded area marks the low-frequency lattice vibrations (< 230 cm^{-1}).

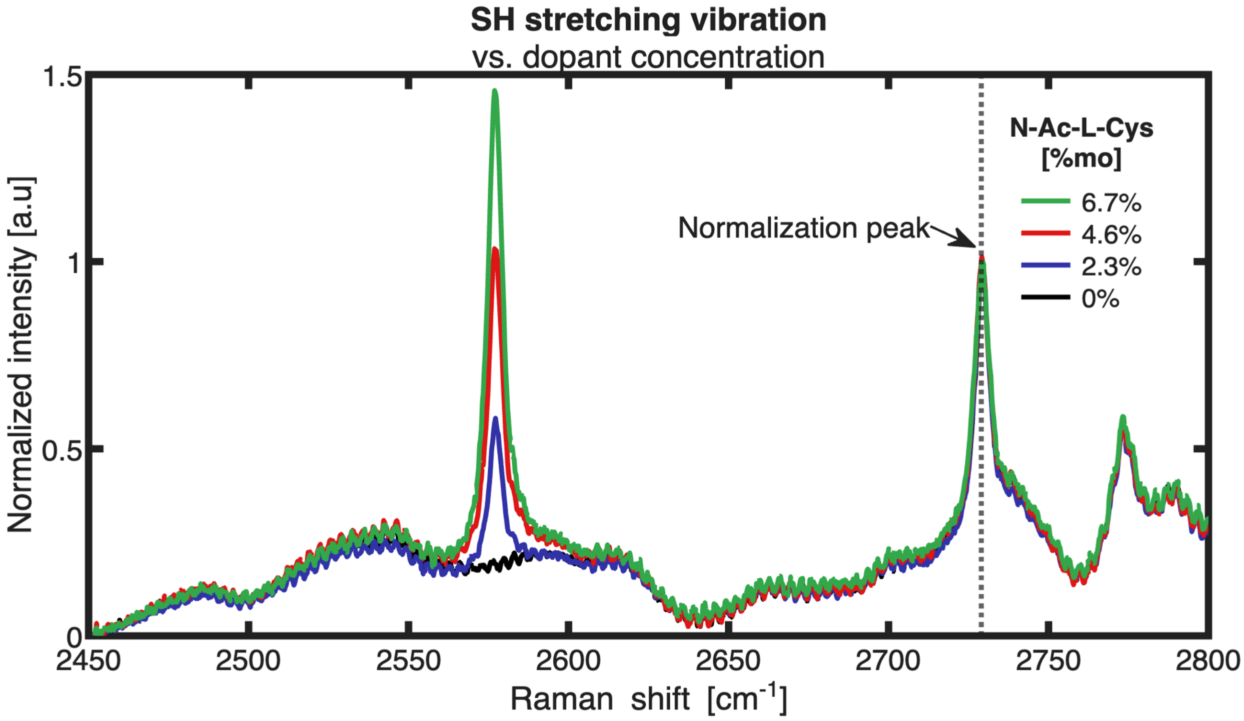

Figure S9: High-frequency Raman spectrum of the SH stretching vibration extended to a range of $2450-2800\mathrm{cm}^{-1}$ demonstrating the high-frequency host peak all spectra were normalized to.

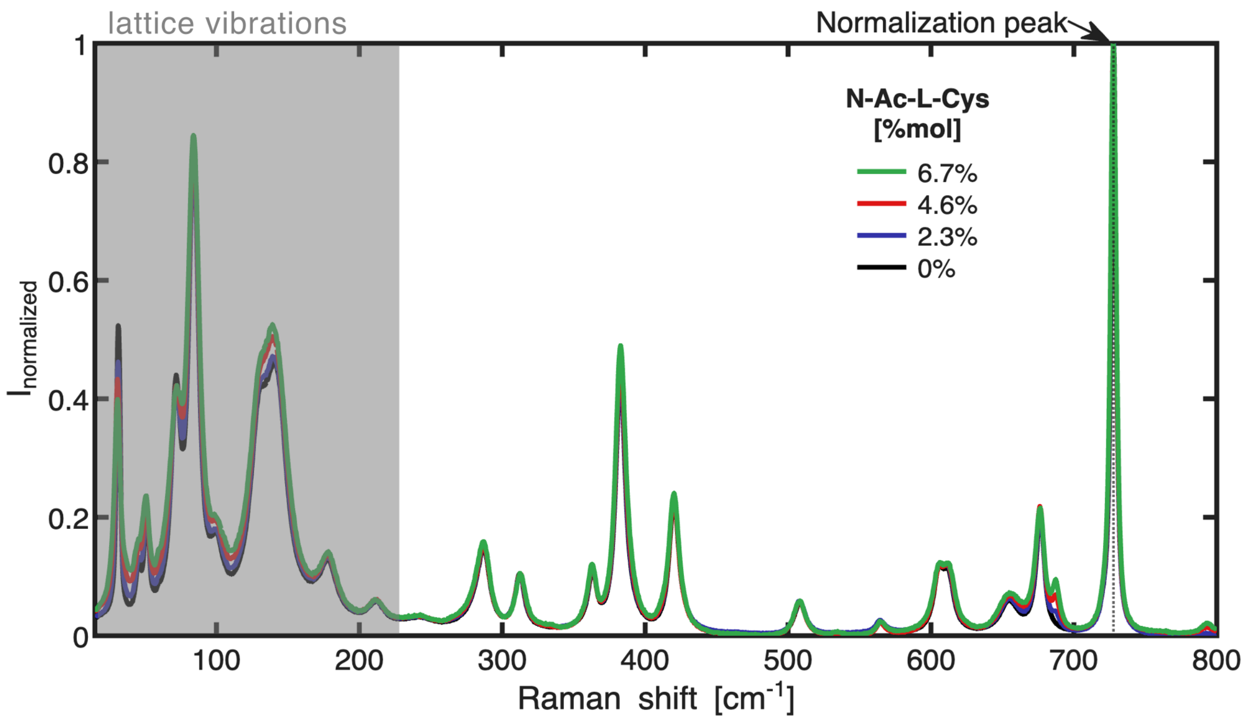

Figure S10: Low-frequency Raman spectrum of the N-Ac-L-Cys doped crystals at various concentrations, between 15 – 800 cm^{-1} demonstrating the high-frequency peak all spectra were normalized to. Grey shaded area marks the low-frequency lattice vibrations (< 230 cm^{-1}).

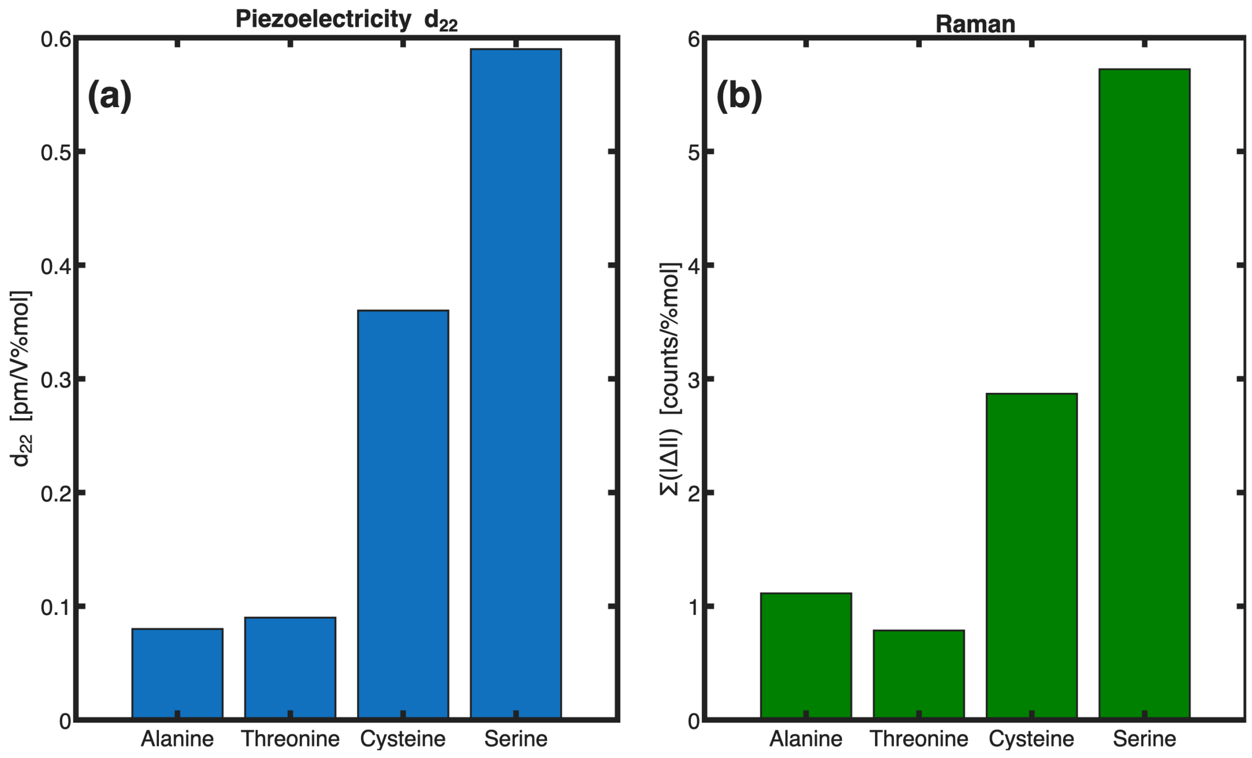

Figure S11: A comparison between the piezoelectricity (a) and $\Sigma(|\Delta I|)$ (b) obtained from the data in Figure 3 in the main text, normalized to dopant concentration measured by LC-MS.

References

[1] G. Kresse and J. Furthmüller, Physical Review B **54**, 11169 (1996).

[2] J. P. Perdew, K. Burke, and M. Ernzerhof, Physical Review Letters **77**, 3865 (1996).

[3] S. Grimme, J. Antony, S. Ehrlich, and H. Krieg, The Journal of Chemical Physics **132**, 154104 (2010).

[4] S. Grimme, S. Ehrlich, and L. Goerigk, Journal of Computational Chemistry **32**, 1456 (2011).

[5] E. Epifanovsky, A. T. B. Gilbert, X. Feng, J. Lee, Y. Mao, N. Mardirossian, P. Pokhilko, A. F. White, M. P. Coons, A. L. Dempwolff, et al., The Journal of Chemical Physics **155** (2021).

[6] R. Resta and D. Vanderbilt, in Physics of Ferroelectrics: A Modern Perspective (Springer, 2007) pp. 31–68.

[7] D. Porezag and M. R. Pederson, Physical Review B **54**, 7830 (1996).

[8] A. Fonari and S. Stauffer, Vasp_raman.py (2013).
